# Use of high doses of second-generation long-acting antipsychotics in the treatment of patients with severe resistant schizophrenia. A mirror-image study

**DOI:** 10.1192/j.eurpsy.2025.499

**Published:** 2025-08-26

**Authors:** F. López-Muñoz, J. J. Fernández-Miranda, S. Díaz-Fernández

**Affiliations:** 1 AGCSM V-Hospital Univ. Cabueñes, SESPA, Gijón; 2 Health Sciencies Dpt., Universidad Camilo J. Cela, Madrid, Spain

## Abstract

**Introduction:**

This study explores whether high-dose treatment with SGA LAIs may benefit patients with schizophrenia who are inadequately controlled on a standard dose

**Objectives:**

The objectives of this study have been to evaluate the retention, effectiveness and tolerability of high doses of second-generation antipsychotic long-acting injectable formulations (SGA LAI) in the treatment of patients with severe resistant schizophrenia.

**Methods:**

A 72-month observational, mirror-image study of patients with severe (CGI-S ≥ 5) resistant schizophrenia receiving treatment with ≥75 mg of risperidone long-acting injectable (RLAI) (N = 60), ≥175 mg of monthly paliperidone palmitate (PP) (N = 60), and ≥600 mg of aripiprazole once-monthly (AOM) (N = 30). All of the patients were previously treated with at least two different APs, with poor outcomes. Patients were eligible if deemed likely to benefit from treatment with SGA LAIs: at risk of medication non-compliance, with a lack of effectiveness, or adverse effects with previous APs. The assessment included the CGI-S, the WHO-DAS, the Medication Adherence Rating Scale (MARS), laboratory tests, weight, adverse effects, reasons for treatment discontinuation, hospital admissions and suicide attempts.

**Results:**

The average antipsychotic doses were: RLAI = 111.2 (9.1 SD) mg/14 days; PP = 231.2 (12.3 SD) mg eq./28 days; and AM = 780 (120 SD) mg/28 days.

Tolerability was good for all LAIs, reducing the side effects reported and the changes in biological parameters compared to previous treatments, especially in the AOM group. Weight and prolactin levels decreased in all LAI treatments; the reduction was statistically significant only among patients treated with AOM (p < 0.05). Two patients discontinued treatment due to side effects with AOM, five with PP and nine with RLAI .

There were four discontinuations with RLAI, two with PP, and one with AOM due to a lack of effectiveness. After three years, the scores decreased in CGI-S (p < 0.01) and in WHO-DAS in the four areas with all injectables. MARS increased with all LAIs (p < 0.01), especially with PP and AOM.

We report a statistically significant decrease in both hospital admissions (p <0.001) and suicide attempts (p < 0.001) at the end of 36-month treatments, compared to the previous three years, without any difference across the three LAIs. In the previous three years, 60 patients discontinued their AP treatment, and 11 during the three-year follow-up (p < 0.0001

**Conclusions:**

Our study indicates the good effectiveness and tolerability of RLAI, PP and AM at high doses. These SGA LAI treatments improved treatment adherence and outcomes of the patients, with good tolerability, helping them to achieve clinical stabilization and better functioning. Therefore, we suggest that, in some illness-critical conditions, high doses of SGA LAIs could represent an alternative to clozapine.

**Disclosure of Interest:**

None Declared

